# Sequencing and phylogenetic analysis of infectious bronchitis virus variant strain from an outbreak in egg-layer flocks in Baghdad, Iraq

**DOI:** 10.14202/vetworld.2020.1358-1362

**Published:** 2020-07-16

**Authors:** Abdullah O. Alhatami, Furkan Alaraji, Husam Muhsen Abdulwahab, Yahia Ismail Khudhair

**Affiliations:** 1 Department of Microbiology, Faculty of Veterinary Medicine, University of Kufa, Iraq; 2 Department of Pathology and Poultry Diseases, Faculty of Veterinary Medicine, University of Kufa, Iraq; 3 Department of Internal and Preventive Medicine, College of Veterinary Medicine, University of Al-Qadisiyah, Iraq

**Keywords:** infectious bronchitis, phylogenetic tree, poultry, real-time polymerase chain reaction, spike 1 gene

## Abstract

**Background and Aim:**

Infectious bronchitis (IB) has an influential economic impact on the poultry industry, causing huge losses each year due to the condemnation of infected chickens. Despite the use of many kinds of vaccines in Iraq, it is common to find IB problems in vaccinated chickens. Information about the strains that affect Iraqi chickens is very limited. Therefore, we aimed to detect the currently circulating strains of IB virus that cause frequent outbreaks in egg layers despite the use of vaccination against the virus.

**Materials and Methods:**

Isolate detection, sequencing, and phylogenetic analysis were performed using a rapid IB virus antigen kit (32 tracheal swabs), flinders technology associates (FTA) card (32 tracheal swabs), and partial gene sequencing (16 positive FTA samples).

**Results:**

The isolated strain was different from other strains, especially the strain isolated in the North of Iraq (Sulemania Strain) and shares 98% homology with an Israeli strain (Israel variant 2, IS 1494).

**Conclusion:**

Although more studies are needed to detect IB virus strains circulating in Iraq, this work lays the foundation for making a good strategy to control the disease and selecting vaccines that should be used in farms.

## Introduction

An important viral disease affecting the poultry industry globally and locally in Iraq is the infectious bronchitis virus (IBV), which causes an acute, highly contagious respiratory disease. The identification of endemic IBV strains promotes better control of the disease and prevents production losses [1]. IBV belongs to the family *Coronaviridae* and the genus *Gamma-coronavirus*. The single-stranded RNA (positive-sense, ~30 kb) of this enveloped virus has several open reading frames [2]. Although the nucleocapsid (N) gene is highly conserved among coronaviruses, the spike (S) glycoprotein (particularly S1) gene contains highly variable components in these viruses [3].

IBV usually is subjected to changes due to mutations and genetic recombination. Therefore, a mutation in the S1 gene may affect virus tissue tropism and virulence [4]. Consequently, IBV can spread widely to affect different regions of the world, creating huge difficulties in controlling the disease due to these newly modified strains [3,4]. Even with high numbers of the globally identified genotypes or serotypes of IBV, cross-protection generated by those strains is barely present [5]. The highly variable S1 gene represents the ideal genetic target for monitoring viral evolution occurring in IBV, especially in strains with high rates of serotype correlations [6]. Therefore, sequencing the S1 gene of IBV strains detected in the field is critical for control programs and epidemiological purposes. The spike protein is a major structural protein of 1162 amino acids that are cleaved into 535-AA-SP1 (S1, N-terminus) and 627-AA-SP2 (S2, C-terminus). The S1 gene induces serotype-specific based antibody neutralization through its two hypervariable regions [7]. Many polymerase chain reaction (PCR)-based techniques have been developed to monitor the prevalence of IBV by targeting and analyzing the S1 gene. Reverse-transcriptase PCR (RT-PCR) and nucleotide sequencing of the S1 gene are routinely used to detect IBV genotype; in addition to real-time quantitative PCR (RT-qPCR), which also is used to identify IBV from field samples. These molecular methods are highly sensitive and specific compared with other diagnostic methods [8-10]. Since its discovery in 1931, and despite massive routine vaccination, IBV continues to be one of the major concerns in the poultry industry worldwide. This may be due to many reasons, one of which is the continuous emergence of new IBV strains [11] with unique genetic determinants identified in each geographical area.

To the best of our knowledge, only one study to date reports the molecular characterization of IBV that resulted in a broiler strain (Sul/01/09), identified in the Kurdistan region of Iraq. Therefore, the current report describes the role of IBV during an outbreak of the respiratory disease in an egg-layer farm in the Baghdad region of Iraq, investigates the genetic characteristics of this field strain by analyzing the S1 gene and compares it with other isolates registered globally for developing significant vaccines to control this disease.

## Materials and Methods

### Ethical approval

This study does not require ethical approval. However, all birds in the current work were treated humanely following International and National criteria of animal care and use.

### Poultry farms

An outbreak occurred during January 2018 in Al-Janoob Company, located in the Al-Wahda district of Baghdad, Iraq. This was composed of eight caged houses of egg layers with a total number of 640,000 hens and 170,000 rearing pullets. The outbreak was not reported to the national authority. All flocks were vaccinated with commercial live-attenuated NOBILIS IB 4-91. Respiratory symptoms began in 28-day-old birds of an ISA Brown flock composed of 88,000 birds per house. Clinically, the symptoms were suspected to be IBV infections. Necropsies were performed, and gross lesions were evaluated.

### Sample collection

Sixty-four tracheal swabs (two swabs per bird) were collected during January 2018, from birds that showed such respiratory manifestations.

### Rapid IBV antigen detection

Thirty-two tracheal swabs were used for serological diagnosis of IBV antigen using a Rapid IBV Ag Test Kit (RG1513DD, Bionote, Korea), which is a chromatographic immunoassay for qualitative IBV antigenic detection in avian swab samples. Tests were performed according to the manufacturer’s instructions.

### RNA extraction

Samples were pooled on flinders technology associates (FTA) cards (Whatman^®^ FTA^®^ card technology) with four sample areas per card (total homogenate volume up to 100 μL) containing chemicals for lysing cells, denaturing proteins, and protecting nucleic acids against damage from nucleases, ultraviolet radiation, and oxidation [12,13].

FTA cards containing viral RNA were outsourced to AniCon Labor GmbH (Muehlenstraße 13a 49685 Hoeltinghausen, Germany), where the extraction of the IBV RNA was performed using Kylt^®^ RNA/DNA Purification Kit through the manufacturer’s protocol.

### Real-time RT-PCR

RT-PCR runs were generated by AniCon Labor GmbH. Briefly, species-specific and variant-specific RT-PCR methods were performed to detect avian coronavirus (aCoV, including IBV) and IBV variants. Hybridization probe-based chemistry was used with the following primers: Kylt^®^ IB-aCoV, Kylt^®^ IBV-Variant 02, Kylt^®^ IBV-Variant 4/91 (793b), Kylt^®^ IBV-Variant Arkansas, Kylt^®^ IBV-Variant D1466, Kylt^®^ IBV-Variant D274, Kylt^®^ IBV-Variant Italy02, Kylt^®^ IBV-Variant Massachusetts, Kylt^®^ IBV-Variant Q1, Kylt^®^ IBV-Variant QX, and Kylt^®^ IBV-IB80 (AniCon Labor GmbH). RT-PCR, CFX96, and CFX384 (Bio-Rad, Hercules, CA, USA) systems were used according to the following conditions: 50°C for 10 min and 95°C for 1 min (initial denaturation), then 42 cycles of 95°C for 10 s, 60°C for 1 min, and read.

### Sequencing of S1 gene products and GenBank accession number

The amplified RT-PCR products were sequenced by AniCon Labor GmbH. Sequences of the Iraqi-IBV-strain S1 gene were deposited in the GenBank database available from the National Center for Biotechnology Information (NCBI) website to collect accession numbers.

### Sequences alignment and phylogenetic analysis

IBV S1 gene sequences obtained herein were compared with sequences of IBV deposited globally in the GenBank database using the NCBI-based BLAST search. The identities of the sequences were analyzed by DNAstar software (DNASTAR, Madison, WI, USA; https://www.dnastar.com/), and Molecular Evolutionary Genetics Analysis (MEGA) X software (https://www.megasoftware.net/) were used to construct the phylogenetic tree.

## Results

### Clinical signs and postmortem examinations

**Figure-1:**
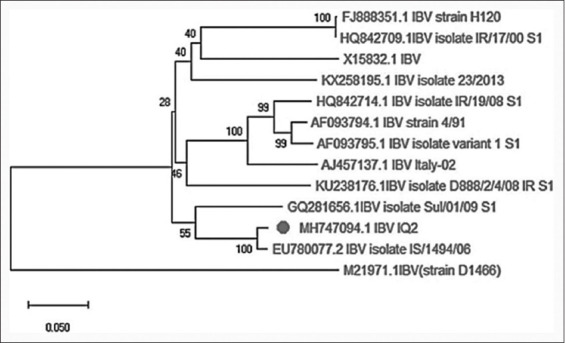
Phylogenetic tree of the infectious bronchitis virus based on the infectious bronchitis virus-IQ2 S1-spike gene partial sequencing with a comparison to world sequence databases.

The flock suffered from signs of infectious bronchitis (IB) disease, and the mortality rates reached 8% for approximately 10 days. Birds suffered the following symptoms: Coughing, sneezing, and rales with depression. Postmortem findings included tracheal congestion, and caseous materials that partially obstructed the trachea and the tracheal bifurcations, and pneumonia and fibrinous airsacculitis (Figure-1). Kidneys were enlarged and congested and contained an accumulation of urate in the nephrons and ureters.

### Rapid IBV antigen detection

Thirty-two (100%) of the examined samples were positive for the presence of the IBV antigen.

### Real-time RT-PCR

Viral RNA from the FTA cards (four spots pooled) with the sample number (A1800428.001) showed positive results with species-specific and IBV-variant-specific RT-qPCR (Table-1).

**Table-1:** Avian coronavirus (aCoV incl IBV) and IB variant detection using species-specific and variant-specific RT-PCR.

Sample no.	A1800428.001
Sample description	FTA-card (4 spots pooled)
aCoV inkl IBV^1^	
Result	Positive
CT	17,1
793b, 4/91, 1/96 and CR88^2^	
Result	Not detected
CT	-
Massachusetts^2^	
Result	Positive
CT	29,9
D1466^2^	
Result	Not detected
CT	-
D274^2^	
Result	Not detected
CT	-
Italy02^2^	
Result	Not detected
CT	-
Arkansas^2^	
Result	Not detected
CT	-
Variant2^2^ (Israel02, IS 1494)	
Result	Positive
CT	15,7
IB 80^2^	
Result	Not detected
CT	-
Q1^2^	
Result	Not detected
CT	-
QX^2^	
Result	Not detected
CT	-

^(¹)^ species-specific Real-Time RT-PCR: Detects Infectious Bronchitis Virus and Turkey Coronavirus, ^(^2^)^IBV variant-specific RT-PCR. IBV=Infectious bronchitis virus, IB=Infectious bronchitis, RT-PCR=Reverse transcriptase-polymerase chain reaction, FTA=Flinders technology associates, aCoV=Avian coronavirus

### Sequencing of S1 gene products and GenBank accession number

A 630-bp and 730-bp fragments of the S1 protein-coding gene were sequenced by AniCon Labor GmbH. The sequences were deposited in the NCBI GenBank under accession numbers MH747093 and MH747094 with the name aCoV strain IQ1 and IQ2 spike glycoprotein (S1) gene, respectively.

### Sequences alignment and phylogenetic tree

The genetic relationship between the S1 gene sequence of the MH747094.1 IBV-IQ2 strain and sequences of vaccine and other virulent strains are presented in Figure-1 and Table-2. The MH747094.1 strain was closely related (100% similarity) to EU780077.2_IBV_isolate_IS/1494/06 (Israeli variant 2), whereas the percentage of sequence identity was 55% compared with GQ281656.1IBV_isolate_Sul/01/09_S1 (Sulaimania isolate). Moreover, the nucleotide similarity was 40% and 28% compared with FJ888351.1IBV strain H120 and AF 093794.1IBV strain 4/91, respectively.

**Table-2:** Percentage of nucleotide divergence of IBV-IQ2 strain in comparison to 12 IBV published isolates calculated by Mega X software.

FJ888351.1_ IBV_strain_H120										
KX258195.1_ IBV_isolate_23/2013	0.21									
HQ842714.1_ IBV_isolate_IR/19/08_S1	0.25	0.23								
HQ842709.1 IBV_isolate_IR/17/00_S1	0.00	0.21	0.25							
GQ281656.1 IBV_isolate_Sul/01/09_S1	0.23	0.18	0.24	0.23						
MH747094.1_ IBV_IQ2	0.19	0.21	0.21	0.19	0.13					
EU780077.2_ IBV_isolate_IS/1494/06	0.21	0.22	0.20	0.21	0.13	0.02				
X15832.1_IBV	0.23	0.24	0.21	0.23	0.27	0.20	0.20			
KU238176.1 IBV_isolate_ D888/2/4/08_IR_S1	0.26	0.23	0.22	0.26	0.21	0.19	0.18	0.24		
AJ457137.1_ IBV_Italy-02	0.27	0.25	0.11	0.26	0.20	0.22	0.22	0.24	0.21	
M21971.1 IBV (strain_D1466)	0.54	0.53	0.51	0.54	0.48	0.51	0.50	0.56	0.53	0.52
AF093794.1_6 IBV_strain_4/91	0.25	0.23	0.06	0.25	0.23	0.21	0.20	0.21	0.20	0.10
AF093795.1_ IBV_isolate_variant_1_S1	0.26	0.24	0.07	0.26	0.23	0.21	0.21	0.22	0.20	0.12

MEGA X program software. in comparison to some selected strains. IBV=Infectious bronchitis virus

## Discussion

IB has an influential economic impact on the poultry industry, causing huge losses each year due to the condemnation of infected chickens [14]. Since IBV was identified during the early 1990s, IBV (4/91 type) has frequently been reported in Europe and many countries around the world [15]. IBV vaccine strains can perform recombination with field strains, reversing virulence [16]. Such characteristics have encouraged verification of the relationship between vaccine and field strains [17]. Although a 793/B-serotype-based attenuated vaccine is available, some 793/B-serotype viruses remain active in Europe and several countries [16], and the live-attenuated or killed Massachusetts (Mass) strain-dependent vaccines are most widely used for vaccination programs throughout the world. Nevertheless, there is an increased failure of IBV vaccination programs, particularly against the 4/91 IBV strain, in addition to the circulation of many IBV-vaccine-related strains [18,19]. Most of the IBV-variant strains have distinctive characteristics due to their global distribution; however, some strains are unique to certain regions, and these properties occur for unknown reasons.

The current work represents the first identifying and genotyping report of IBV that resembles the Israeli isolate in Iraq. The results of the sequencing and phylogenetic tree analysis indicate that our isolate shares 98% homology with the Israel variant 2 (IS 1494). In 2011, Zana *et al*. [20] reported a newly isolated strain along with other regionally identified isolates from other Israel strains (IS/720/99, IS/885), and Waleed *et al*. [21] also registered strains 793/B and Mass from infected broilers in the South of Iraq. Thus, isolates from different parts of Iraq demonstrate large differences in homology. In addition, in the Sulaimaniah isolate (Sul/01/09), the birds displayed nephron pathological lesions, and the virus was detected from kidney samples but not from tracheae. Therefore, the Sulmania (Iraqi strain) that affected broilers differs genetically from our strain that infected layers. Furthermore, our isolate shared <82% and 79% in nucleotide sequence with vaccine strains FJ888351.1 and KX258195.1, respectively, which may explain the occurrence of infection despite vaccination.

Therefore, our work lays the main foundation including RNA-based S1 protein transcript sequencing and the related phylogenetic analysis, to initiate launching strategies for control of IBV in the field.

## Conclusion

Despite the intensive vaccination program in Iraq, IB outbreaks have occurred for many decades. In this study, we isolated a strain of IBV very different from that of local North Iraq (Sulemania Strain) but similar to an Israeli strain (Israel variant 2, IS 1494). Genetic characterization of the Iraqi circulating IBV strains is critical because of inadequate data from this location. More studies revealing such strains will pave the way for a good strategy of controlling the infections and understanding or developing types of vaccines that should be used in the future.

## Authors’ Contributions

AOA and HMA visited the infected farm, collected the samples, and performed the rapid IBV antigen detection. FA and YIK visited the infected farm, collected the samples, run the rapid IBV antigen detection, confirmed the results using FTA card that was sent to Germany, deposited the genetic information into the NCBI database, analyzed the differences between the strains of the virus, and drafted the manuscript. All authors revised the manuscript. All authors have read and approved the final manuscript.
